# Herd-Level Risk Factors for Bovine Tuberculosis: A Literature Review

**DOI:** 10.1155/2012/621210

**Published:** 2012-06-28

**Authors:** Robin A. Skuce, Adrian R. Allen, Stanley W. J. McDowell

**Affiliations:** Veterinary Sciences Division, Agri-Food and Biosciences Institute, Belfast BT4 3SD, UK

## Abstract

Bovine tuberculosis (TB), caused by *Mycobacterium bovis*, is one of the most challenging endemic diseases currently facing government, the veterinary profession, and the farming industry in the United Kingdom and Ireland and in several other countries. The disease has a notoriously complex epidemiology; the scientific evidence supports both cattle-cattle and wildlife-cattle transmission routes. To produce more effective ways of reducing such transmission, it is important to understand those risk factors which influence the presence or absence of bovine TB in cattle herds. Here we review the literature on herd-level risk factor studies. Whilst risk factors operate at different scales and may vary across regions, epidemiological studies have identified a number of risk factors associated with bovine TB herd breakdowns, including the purchase of cattle, the occurrence of bovine TB in contiguous herds, and/or the surrounding area as well as herd size. Other factors identified in some studies include farm and herd management practices, such as, the spreading of slurry, the use of certain housing types, farms having multiple premises, and the use of silage clamps. In general, the most consistently identified risk factors are biologically plausible and consistent with known transmission routes involving cattle-cattle and wildlife-cattle pathways.

## 1. Introduction

Bovine TB is a chronic disease of animals caused by infection with the slow-growing, obligate intracellular bacterium *Mycobacterium bovis *[1, 2]. This highly adapted and “successful” pathogen has a world-wide distribution and in several countries bovine TB remains a major, costly infectious disease of cattle and other domesticated, feral and wild animal populations, including badgers, possums, deer, goats, sheep, and camelids [3–5]. Bovine TB is an OIE (World Organisation for Animal Health) listed (formerly List B) disease [6, 7].

Bovine TB affects cattle health, impacts negatively on profitability and trade, and can decimate years of genetic improvement towards desirable production traits [8]. It also impacts negatively on the welfare of affected farming families [9]. Although effectively controlled by herd testing, milk pasteurization, meat inspection, health surveillance, and BCG vaccination, transmission to humans can occur and is still considered a public health risk [10–12], although some more recent opinion considers this risk to be negligible [13]. Hence, bovine TB control is currently more concerned with trade implications.

Despite sustained and costly implementation of eradication programmes since the 1950s bovine TB has not been eradicated from either the United Kingdom (UK) or Ireland. Indeed, there has been a sustained and largely unexplained increase over the last 25 years in parts of the UK [14]. Consequently, bovine TB is the most complex and difficult multi species endemic disease currently facing government, the veterinary profession, and the farming industry in the UK and Ireland [15, 16]. Bovine TB epidemiology, in the UK at least, is exceptionally complicated, and the relationship between evidence, uncertainty and risk has been difficult to communicate [17]. It is recognised as a very significant policy challenge and continues to be almost inevitably highly politicized [18].

Pathogenesis studies suggest strongly that the route of transmission of bovine TB is largely *via* the respiratory system, requiring transmission *via* infectious aerosols [19]. Consequently bovine TB is principally a respiratory infection and the majority of infections are thought to occur *via* “direct” aerosol transmission between animals in close proximity. Although considered to be of lesser importance, oral ingestion of mycobacteria from farm environments cannot currently be excluded [1]. Whilst it is important to view bovine TB as an infectious disease which requires preventive as well as control measures, *M. bovis* infection in cattle now rarely presents as clinical disease. More commonly it appears as apparently healthy animals responding to an immunological test based on tuberculin, an entirely different scenario to that which existed when control programmes were first introduced [20].

In countries with advanced test and control programmes (a comprehensive set of surveillance and control measures to address cattle-cattle transmission) bovine TB tends to be a low-incidence infectious disease with an apparently low transmission rate. Infection appears to be relatively poorly transmitted between cattle in most, but not all, circumstances.

## 2. Herd Testing and Management

Monitoring of the cattle population for *M. bovis* infection depends on national programmes of herd tuberculin testing, supported by active abattoir surveillance [1, 12, 21]. The frequency of such testing is determined by the recent local incidence, ranging from annual testing (in Northern Ireland and other parts of the UK and Ireland) to four-year testing (in parts of England) [1, 21]. Limited tuberculin test sensitivity is also likely to have contributed to under- or over-estimation of the impact of several risk factors, such as, cattle contact and movement in some studies. To compound the above, multiple unreported local cattle movements and contacts are described between farms in several studies and are recognised as a factor in underestimating the role of contact and movements, particularly over short range.

Early diagnosis and intervention to interrupt transmission are the priority for control and the effectiveness of testing and removal of infectious animals will impact on transmission and depends on: how early the infections are detected, how sensitive the test(s) actually are in practice and other variables, including interoperator characteristics, test interval, and/or time to derestriction. More severe tuberculin test interpretation and/or supplementary immunological tests (cell-mediated immunity and/or humoral immunity) may be applied in defined circumstances. The herd is placed under movement restriction until all animals clear two short-interval tuberculin tests. Animals may also test “inconclusive” to the tuberculin test, a proportion of which test negative at the next short-interval test but have since been shown in an Irish study to be 12 times more likely to be TB positive at the next test than other animals [22]. Between 11.8% and 21.4% was confirmed positive at the laboratory, compared to 34–39% for standard tuberculin reactors. In summary, inconclusive reactors are at increased future TB risk [22, 23], whether they clear the next test or not and this is now reflected in policy options.

Bovine TB “reactors”, and on occasions the exposed cohort, should be removed quickly from affected herds and contact tracing and testing should be implemented. Whole-herd depopulation may be indicated on rare occasions. The impact of such whole herd depopulation, for either bovine TB or bovine spongiform encephalopathy (BSE), on the recurrence of bovine TB was investigated in a recent Irish study [21]. Recurrence may reflect residual infection in cattle and/or reinfection from other sources, potentially including contiguous spread, acquired infection, infectious wildlife, and environmental contamination and may also be a consequence of imperfect test sensitivity [24]. It was concluded that future risk varied significantly by previous TB risk and reason for depopulation and that whole herd depopulation was effective in reducing future bovine TB risk. The BSE depopulated herd results were similar to a recent GB study [25] where whole herd depopulation for foot-and-mouth disease (FMD) was also not associated with reduced future bovine TB risk.

Key to understanding bovine TB epidemiology is the relationship between infection and disease (TB) and the relationship between disease and transmission. Hence, it is important to consider those risk factors, such as, infectious contacts between animals and their movements, which theoretically facilitate such transmission. The identification of risk factors and risk settings for infection and transmission is also intimately linked with those factors which affect susceptibility.

## 3. Risk Factor Studies

Infectious diseases in general arise from an interaction between the infectious agent, the host, and a range of covariables, which may include other infectious diseases and the environment. Risk factors (biological, behavioural, environmental, or genetic) are known to influence both transmission and susceptibility. They may operate at different scales; regional-level, herd-level, and animal-level and may vary across regions due to factors, such as, differing farm structures, farm management practices, bovine TB control and eradication programmes, regional TB incidences, wildlife densities, and the relative importance of specific risk factors by area.

The risk of a bovine TB episode is accepted to vary between herds with some herds experiencing multiple breakdowns over time, whilst others appear to remain free of infection. Also, the nature of bovine TB breakdowns is not uniform; they can be classified as “sporadic”, “persistent”, “recurrent”, and so forth and the literature supports the view that different risk factors are likely to apply, almost on a case-by-case basis. However, some risk factors have tended to emerge in several published studies and we have discussed these here.

It is important to note that epidemiological studies may differ in the variables examined, the exact measures used (in relation to the association with wildlife, etc.), and the study size and power. Therefore, not all risk factors would be expected to be identified equally across all studies. Those risk factors which tend to converge from disparate studies would support the currently hypothesized sources of infection and routes of transmission.

There are no guarantees that such studies would identify significant risk factors, although the fundamental premise of epidemiology is that disease occurrence in populations is not random but rather is associated with identifiable factors. Not all of the studies summarized below are classical “risk factor” studies; instead they are observational studies and it is important to remember that risk factors identified in herd- and animal-level studies are *associated* with the measured outcome and are not necessarily causal. Some studies include analysis of wildlife-related risk factors, which have not been discussed in any detail here.

Summaries of the studies assessed as part of this paper have been included below in two groups. The first are classical case-control studies, the second are predominantly cohort-based studies or case-control studies focused on specific risk factors. Most of the studies listed under “other epidemiological studies” used preexisting data (e.g., cattle movement and bovine TB test data) and have addressed specific epidemiological questions, such as, the role of cattle movement.

Previous (case-control) studies, mostly in the UK and Ireland, have identified a number of risk factors associated with TB herd breakdowns. These include the purchase of cattle [26–28], the occurrence of TB in contiguous herds, and/or the surrounding area [29, 30] as well as herd size [28, 30]. The most frequently identified risks for herd-herd transmission included cattle movements and trading, where general trading or purchase from markets or herds in hot-spot areas or from infected herds have all been linked with increased risk for the receiving herd [31].

The following are among the risk factors shown to influence the potential of direct and indirect (*via *faeces and urine) exposure of cattle to the wildlife reservoir (badgers in the UK and Ireland) when at pasture; stocking regime (set-stocking), rotational *versus* strip grazing, stocking densities, farm habitat types, and livestock production intensity [31]. Sharing feed or water between cattle and wildlife when housed or at pasture, housing type and storing manure indoors are associated with the differential risk of transmission between cattle and wildlife. Results in relation to the association with the presence of badger setts or local badger density have been more variable with some studies reporting an increased risk associated with the presence of badger setts on the farm [26, 29] or local badger density [4] while others have found no association with the presence of badger setts either on the farm or the surrounding area [27, 30].

Other evidence, and in particular observational studies on badgers in GB, has suggested possible routes of transmission from badgers to cattle [32], including the possibilities of direct contact between cattle and badgers at pasture, indirect contact between cattle and infected badger faeces, urine and wound discharges, the likelihood of which is increased where cattle can access badger setts and latrines, and/or where badgers access cattle feed and/or water troughs. There is also substantial evidence in GB of badger visits to farmyards and buildings to access a range of feed sources including cattle feed in stores, maize silage, and accessing feed in troughs [33, 34], all of which would increase the likelihood of both direct and indirect transmission of infection. The physical exclusion of badgers from farm buildings has been suggested as the simplest and potentially most effective method of reducing contact between badgers and cattle [32], although issues of practicality, likely farmer uptake and compliance, require further investigation.

Many farm and management variables are likely to be highly correlated with factors, such as, herd size, herd type, and to a lesser degree herd location. Herd size, for example, was a risk factor in a number of previous studies, although it is unclear whether this variable is acting as a risk factor *per se*, as a partial summary measure of other factors or because of inherent changes in the herd level sensitivity and specificity of the test as herd size increases.

Other factors identified in some studies include farm and herd management practices, such as, the spreading of slurry [26]; the use of certain housing types [27]; farms having multiple premises [27]; the use of silage clamps [28]. In general, the most consistently identified risk factors are biologically plausible and consistent with known transmission routes involving cattle to cattle and badger to cattle pathways. Whilst many of the general risk factors for the introduction and spread of bovine TB have been identified, less is known about the practical measures that herd keepers could reasonably take to minimize their risk and the possible impact of common biosecurity practices.

## 4. Case-Control Studies

In bovine TB, several risk factors (e.g., cattle husbandry and environmental practices) have been suggested as predisposing farms to TB breakdowns [35]. However, they are not amenable to experimental investigation due to the large number of variables, the impracticality and cost of conducting controlled experiments on commercial livestock farms, and the need for data from a large number of representative bovine TB breakdowns. In such circumstances, a “case-control” study provides the appropriate approach [1].

The essence of a case-control study is firstly to identify if specified risk factors are statistically associated with the occurrence of a disease or condition, while controlling for confounding and interaction and secondly to estimate the magnitude of any such risk. Within a case-control design, further options exist as to whether or not cases and control should be matched. The typical output of a case-control study is a list of risk factors associated with the disease, accompanied by the statistical significance of each, the odds ratio (OR) for each, and the 95% confidence interval (CI) for the OR. For example, the study may show that a farm that purchases cattle is twice as likely to experience a bovine TB breakdown as one that does not.

The accompanying statistical significance indicates the likelihood of such a finding not being true (i.e., that such a finding has arisen in the study purely by random chance) and therefore the weight that can be applied to the finding. The 95% CI of an odds ratio indicates the likely range of the predicted risk. An estimated OR of >1.0 indicates that the factor is associated with an increased risk of a breakdown, and the greater the numerical value of the OR, the greater the risk. By contrast an OR <1.0 suggests that the factor reduces risk and is “protective” in relation to bovine TB breakdowns.

A case-control study of risk factors for bovine TB in Northern Ireland was undertaken, based on tuberculin test reactors identified between 1990 to 1992 [29]. The study involved 427 dairy farms (excluding farms with fewer than 30 cattle and herds with reactors in purchased cattle). Variables investigated included the number and nature of farm boundaries, the number of neighbours and their bovine TB history, the number of hedgerows, the presence of badger setts, whether badger carcases had been found on the land, and the possible presence of deer. Two factors were significantly associated with bovine TB breakdowns; the presence of badger setts or carcasses on the farm (OR 2.06, 95% CI 1.27–3.33) and contiguous neighbours with confirmed bovine TB (OR 2.44, 95% CI 1.55–3.86).

A matched case-control study was undertaken in the Republic of Ireland to provide information on the role of farm management practices, environmental factors, and farmer characteristics in the epidemiology of bovine TB. Eighty dairy herds with chronic bovine TB were compared with the same number of herds which had been free of the disease for many years. A standardized questionnaire was used. The study was conducted from August to October 1990, in Counties Cork and Kilkenny. Factors which were identified as possibly contributing to recurrent outbreaks of TB included nutritional factors, cattle purchases (especially bulls), the presence of badgers, and the spreading of slurry. Overall, the findings suggested that intensively managed dairy herds were at greater risk of bovine TB outbreaks than were other herds [26].

Subsequently, a case-control study of 200 herds from East Offaly (Ireland), with cases defined as outbreaks of bovine TB detected at herd test, was undertaken [30]. Herd-level risk factors significantly associated with an increased risk of infection were herd size and the presence of TB in a contiguous herd. Differences between animal types (increased risk in cows, heifers, and bullocks compared to calves) and a reduced risk (protective) in animals purchased since the preceding herd test were found at the animal level. No significant differences were found between cases and controls in the distance to the nearest badger sett or the nearest main sett.

Herd-level risk factors for bovine TB breakdowns based on cattle farms enrolled within the GB Randomised Badger Culling Trial (RBCT), prior to the Foot-and-Mouth Disease (FMD) epidemic in 2001, were investigated [27]. The study (the TB99 study) comprised 268 farms from SW England, with questionnaires on farm management practices completed by staff from the local Animal Health Office. The strongest factors associated with an increased TB risk were movement of cattle onto the farm from markets or farm sales, operating a farm over multiple premises and the use of either covered yard or “other” housing types. Spreading artificial fertilizers or farmyard manure on grazing land was associated with a decreased risk. The presence of an active badger sett mapped to either the farm land or to within 1 km of the farm boundaries was not statistically significant.

Risk factors have been investigated in case-control studies in Europe and the USA. Historical incidence was a robust predictor of the rate of future breakdowns in UK and Irish herds, suggesting that the disease source was not adequately removed, or that some other factor(s) made them particularly susceptible. Herd size was repeatedly identified as a major risk in many studies. Large herds tend to graze larger areas and may purchase and move more cattle, increasing the probability of having contiguous herds that facilitates cattle-cattle spread. The higher production stress of intensive management has been associated with increased risk [26]. Herd breakdowns tend to recur, especially in larger herds, possibly as a result of failing to clear the source and contact with contiguous herds and infectious wildlife.

Larger herds are more likely to have at least one cow with disease. As herd size increases, the probability of at least one case increases and herds of different sizes are therefore at different risks. The observed size distribution of bovine TB-affected herds suggests that animals pose identical risks. Cattle living in different parts of the UK and Ireland probably experience different risks, and there was no consistent indication in the GB TB99 and CCS2005 data to suggest that the presence of any wildlife species, or indeed domesticated species, was associated with the risk of multireactor breakdowns [1]. There is evidence that increasing herd size for financial gain may actually contribute to increased bovine TB incidence [33].

Mathews et al. [4] examined the association between farm habitat features and other factors and the risk of bovine TB in two areas in West and SW England and involved 120 dairy herds in total (excluding herds with bovine TB breakdowns due to imported cattle). Badger road-kill records within 1 km and 5 km proximity of the farm were used as a proxy measure of badger density. The predictors found to be significant included farmland habitat, topography, and indices of badger density and herd size.

A comparative case-control study in England in which risk factors in herds with transient or persistent TB breakdowns were compared to a common set of control herds (229 herds in total) was reported [28]. Interviews with herd-keepers were conducted (March 2000–February 2003). Data on farm management practices were obtained from on-farm questionnaires, whereas the presence of badger setts and the type of habitat cover was determined by field survey of the relevant farms. The purchase of cows was a risk factor for both transient and persistent breakdown. The purchase of >50 cattle and the storage of manure for ≥6 months were risk factors for transient breakdowns, whereas the use of silage clamps increased the risk of persistent breakdown. Rather counter intuitively, decreased odds of both transient and persistent breakdown were associated with higher stocking densities (>3 cattle/ha). Running mixed herd enterprises compared to beef only or dairy only was an additional protective factor against persistent breakdown. Herd size and tuberculin testing interval were also significant risk factors for both transient and persistent breakdowns, whereas active badger sett density and regional location only affected the risk of persistent breakdowns.

Johnston et al. [31] reported the results of a matched case-control study (218 of 401 herds available for analysis) in 4 regions of England and Wales in 2005/2006, where case herds had confirmed infection. The significance of association with risk factors varied clearly by location. Overall, they report that contacts with contiguous herds (OR = 2.24), sourcing cattle from herds with a recent bovine TB history (OR = 1.90), operating a fragmented farm (OR = 2.41), feeding cattle inside housing (OR = 4.89), and presence of dead badgers on farm (OR = 3.10) were all associated with increased risk of a confirmed breakdown. Case herds were more likely to source cattle from herds with a breakdown within the last 2 years and more likely to have more direct contacts with contiguous herds with more confirmed breakdowns in the previous 2 years among contacted herds. They were also more likely to report finding dead badgers on farm. Providing feed outside of cattle housing was protective (OR = 0.41), as was the practice of not providing shelter at pasture for cattle, which may reduce the opportunities for cattle-cattle contacts. Grazing the whole pasture was associated with increased risk, possibly due to the increased potential for badger-cattle contact at pasture. They concluded that there was an increased local risk related to the occurrence of breakdowns amongst neighbours and/or contacted herds and possibly shared exposure to an external source, such as, wildlife. Risk factors tended to vary by region, so control recommendations should reflect local risk.

## 5. Other Epidemiological Studies

Using the GB Cattle Tracing System and VetNet data Brooks, Pollock and Keeling [36] examined the relationship between herd size and persistence of bovine TB on farms. Using a measure similar to the Critical Community Size, the VetNet data revealed that herd size was positively correlated with disease persistence. Carrique-Mas et al. [25] analysed cattle movement data and herd TB history in approximately 4,200 herds, which were restocked after-FMD. Three risk factors were identified in the study; sourcing cattle from herds that were routinely tested for bovine TB more than biennially, a history of TB breakdowns in the restocked farm (1997–2000), and increasing herd size.

Although by design a case-control study, a GB study was undertaken at the animal level and specifically examined the relationship between the selenium, copper, and vitamin B12 status of cattle, and bovine TB infection [37]. The animals involved were 200 reactors and 200 in-contact animals, selected from herds in England and Wales. The study found that lower levels of GSHPx (Selenium) and higher levels of copper were associated with an increased risk of confirmed bovine TB, but there was no association with vitamin B12.

Gilbert et al. [14] assessed the role of cattle movements in the spread of bovine TB in GB using movement records from the Cattle Tracing System data archived. Their study showed that cattle movements, particularly those from areas where bovine TB was reported, consistently outperformed environmental, topographic, and other anthropogenic variables as the main predictor of disease occurrence. Gopal et al. [38] reported on the introduction of bovine TB to NE England by bought-in cattle. Their study investigated 31 herds that experienced confirmed breakdowns between January 2002 and June 2004; nine of which had restocked after-FMD 2001. In all but one of the breakdowns the most likely source of infection was one or more purchased animals. In 17 of the breakdowns, reactor animals were traced to herds from which the same *M. bovis* genotype (spoligotype-VNTR profile) was isolated, and in five breakdowns a different genotype was isolated. Reactors in five of the breakdowns included homebred and purchased animals, providing evidence for the likely spread of the disease by cattle-cattle transmission within the herds on arrival. The lack of geographical clustering of molecular types pointed to the overwhelming source of infection being purchased cattle.

Green et al. [39] used cattle movement data to construct an individual (premises-) based model of bovine TB spread within GB, accounting for spread due to recorded cattle movements and other causes. Outbreak data for 2004 were best explained by a model attributing 16% of herd infections directly to cattle movements, with a further 9% unexplained, potentially including spread from unrecorded cattle movements. The best-fit model assumed low levels of cattle-cattle transmission. The remaining 75% of infection was attributed to local (wildlife and cattle) effects within specific high-risk areas. Green and Cornell [40] investigated herd breakdowns in four counties of England and Wales using data from the national database of bovine TB testing history (VetNet). Factors that influenced herd breakdown included calendar time, herd size, number of cattle tested, the test type, the intertest interval, and spatial grouping of farms.

The proximity of farms to badger setts was compared between Irish farms that had experienced a TB breakdown and those that had not, over the 6-year period from 1988 to 1993 [41]. The data were derived from badger removal in East Offaly, which began in 1989 and continued through 1993. By the end of 1990 approximately 80% of all badgers caught in the 6-year period had been removed. The risk of a multiple reactor TB breakdown decreased for herds at least 1 km away from an infected badger sett and increased as the number of infected badgers *per* infected sett increased. Despite the significantly reduced risk of a breakdown with increasing distance from infected badger setts, the relationship was not strong (sensitivity and specificity of the model were in the low 70s%) and explained only 9–19% of bovine TB breakdowns.

A retrospective cohort study with bovine TB test data [42] investigated breakdown severity as a predictor of future herd breakdowns in Ireland. The hazard (risk) of a future bovine TB breakdown increased directly with number of cattle in the herd, a positive history of previous bovine TB in the herd, and the local herd prevalence of bovine TB. The presence of confirmed bovine TB lesions in reactor cattle was not predictive of the future breakdown hazard when the effects of other factors were controlled.

The Irish study above contrasts with a recent GB study, which showed that ~30% of herd breakdowns extend for >8 months [43] and consume disproportional resources as well as acting as ongoing sources of infection. Breakdown duration was a function of infection status and test performance. Potential explanations for *persistent* infection included suboptimal performance of the bovine TB tuberculin test, delay in its application, or reintroduction of infection. Skin test sensitivity has been estimated at 75.0–95.5% [12]. If the sensitivity was substantially lower [24], failure to detect and remove infected animals would create potential for within-herd persistence and onward spread.

Factors associated with breakdown *recurrence* in Ireland, where detailed animal-level data were available [44], included slurry spreading, purchase of bulls and cattle, presence of inconclusive reactors in the breakdown, and presence of badgers and nutritional status. Where only population-level surveillance data were available, factors associated with recurrence included herd size, reactor number, and recent herd bovine TB history [44–46].

In the DEFRA SE3230 research project (The Problem TB Herd: characterisation, prediction and resolution) breakdown confirmation status was by far the strongest risk factor for persistence (OR = 12.6). They used an improved case definition and concluded that this strong association may best be explained by the tendency to deploy severe interpretation of the tuberculin test in herds with confirmed status and the possibility that true prevalence was underestimated [43]. Their model could predict earlier those herds most likely to sustain persistent infection. Resources and earlier intervention could be directed at those herds. The model predicted that stopping animal movements onto the farm during the breakdown and moving salt licks indoors were associated with a small decreased risk. It is also plausible that a number of the unconfirmed herds were not actually infected.

Analysis of the GB CCS2005 epidemiological data identified that despite increased testing during and after breakdowns ~21% of breakdowns recurred within 12 months. 60% of these recurrences was disclosed at the 6-month followup, suggestive of within-herd persistence. 38% recurred within 24 months [45]. Factors associated with *recurrence* were reactor number and recent history of bovine TB in the herd, consistent with previous studies in Ireland and Northern Ireland [44, 46]. However, they found a lack of association with the confirmation status of the initial breakdown. They conclude that their data support a higher prevalence of infection than observed, residual infection, or repeated reinfection. The main risk factors associated with recurrence in this study ranked as follows: use of “other housing types” (OR = 4.6), number of contiguous farms (OR = 3.2), and borrowing animals (OR = 2.1) [45]. Protective factors associated with decreased risk of recurrence included the presence of rough grass/moorland (OR = 0.3). These recurrent breakdowns may have been reinfected from a local source, such as, wildlife or from cattle movements into the herd. As well as consuming disproportionate resources, the existence of recurrent breakdowns suggests that such herds cannot reliably be cleared of infection and undermines stakeholder confidence in the TB testing programme. They concluded that certain farm practices or characteristics may predispose to reinfection and that a combination of factors was associated with recurrence, rather than just one strong factor.

Abernethy et al. [46] used comprehensive animal-level test and movement data to investigate the effect of selected risk factors on recurrence of bovine TB in breakdown herds after derestriction in Northern Ireland. Factors associated with an increased risk included the number of reactors at the disclosing test, the number of reactors at follow-up tests, the number of follow up tests, the level of bovine TB in the district council area, herd size, the number of cattle purchased during the postoutbreak interval, and a history of bovine TB breakdown(s) within the previous two years.

Despite variation between farming practices within the British Isles, reactor number and recent history of bovine TB were consistent risks for recurrence in Irish, Northern Irish, and GB studies [45]. Whether the breakdown was confirmed or not was the major factor in the duration of breakdowns (persistence) in GB [43] but was not a factor in risk of recurrence and neither was herd size nor cattle movements [45]. This illustrates that the risk factors for different types of breakdown (sporadic, persistent, recurrent, etc.) may well be different. Either way, this could increase transmission potential to local wildlife or to local or more distant cattle herds through cattle contacts and movements, during periods when movement restrictions are not applied. The relative contribution of persistence *versus* reintroduction to recurrence is unknown [45] and although their wildlife data were relatively weak, no association was detected between badger presence and recurrence at the 6 month follow-up herd test. To increase the detection of exposed/infected cattle within herd, there have been suggestions to increase the between-test interval and the duration of herd restriction.

Olea-Popelka et al. [47] attempted to estimate the levels of badger exposure for cattle and to test the hypothesis that increased badger exposure does not increase the risk of bovine TB in selected Irish herds. They used data from the Four Areas Trial badger cull in Kilkenny (1996–1999). The specific location of cattle within each farm, and the length of time that cattle spent in each farm field during the grazing season, and in the barnyard during winter, was used to build an exposure coefficient to quantify the amount of badger exposure that cattle encountered either on pasture or in the barn. The study design was a matched case-control study in which the control herds were selected using incidence density sampling. During the 4-year study period, 543 badgers were removed and of those 96 badgers were bovine TB positive and 96 herd breakdowns occurred. There was a significant association between case herds and having a higher badger sett exposure coefficient during 1996–1998, but no significant association between case herds and having a higher exposure coefficient based on the number of badgers, or the number of bovine TB-positive badgers, during September 1997–December 1999 was found. It would be valuable to take the same approach to quantifying within-herd cattle contacts in housing and at pasture.

Porphyre et al. [48] investigated risk factors for bovine TB on New Zealand cattle farms and their relationship with possum control strategies. Study design was a retrospective cohort based on data obtained from the TB testing surveillance programme. The model showed that, despite intensification of possum control strategies over time, proximity to forest parks (a principal possum habitat in this area) remained a significant predictor of the number of confirmed cases of TB detected *per* farm *per* year. Their analyses showed a significant, threefold increase in bovine TB risk in dairy cattle relative to beef, conditional on the size of the local possum habitat. Other factors identified included the cattle population size and the presence of previous infection.

Ramírez-Villaescusa et al. [49] examined herd- and animal-level risks associated with bovine TB tuberculin test positivity in cattle in 148 herds in RBCT areas of SW England. Data on cattle on these farms were sourced from the bovine TB VetNet database from 1996 to 2004 and from the British Cattle Movement Scheme database. Results showed that cattle were more likely to react to the bovine TB tuberculin test when they had been present at a previous bovine TB herd test(s) where other cattle had reacted. This positively correlated with age and number of tests. Cattle on restocked farms were less likely to react to the tuberculin test compared with cattle on continuously stocked farms. These results highlight the likely importance of exposure to infected cattle present at a previous test as a source of infection to cattle that subsequently became reactors. This suggests that there was a lower risk of exposure to bovine TB to cattle in newly formed herds.

Further analysis [50] examined herd and individual animal risks associated with tuberculin test positivity. Farms restocked for <12 months after-FMD had a significantly reduced risk compared to continuously stocked farms. The feeding of mineral licks and vitamin supplements was associated with reduced risk. Storing manure and slurry indoors or in a closed container, spreading manure all year, possession of dairy cattle, increased herd size, and purchase of cattle from markets as well as farm location were associated with increased risk. The authors concluded that whole herd removal might have reduced the infectious load on these premises, but this did not continue once cattle were reintroduced. The method of slurry storage or spread might has allowed *M. bovis* to persist in the environment in some cases. The increased risk associated with cattle purchase and continuous stocking *versus* restocking supports a role for undetected infection in cattle as a risk to other cattle.

## 6. Farm-Scale Studies

Most of the studies outlined above operate over quite large areas, larger than the individual farm scale, where model predictions would be even more useful. However, a recent study, using GB RBCT data from one year into treatment to one year after treatment, presents an analysis of spatial farm level, herd-based risk factors associated with the probability of a confirmed bovine TB breakdown [51]. Within reactive and survey-only areas, the risk of a confirmed bovine TB breakdown was associated with two factors; increasing numbers of active badger setts and having cattle herds within 1.5 km. For proactive areas, the strongest predictor of bovine TB risk was the number of *M. bovis*-positive badgers culled initially within 1.5 km, suggesting that a risk remained for those herds, which was not removed by badger culling. They provide further evidence that the local infection in cattle and badgers is linked. Within the RBCT data they found that dairy herds were more at risk than beef herds and tended to rely on one particular breed of cattle, whereas beef farms tended to use a mixture of breeds and crossbreeds. Whilst acknowledging the complex interaction of risk factors, they indicated that a breed effect might operate [52].

Further, recent, mixed modelling and event history analysis were used to investigate individual risk factors in RBCT data analysis, again at the individual farm level. Farm characteristics, in particular herd and farm size, number of land parcels and being contiguous to other breakdowns were significant and consistent risks. They also identified increased risks for those herds subjected to reactive badger culling and those with increased herd size and increased and fragmented farms [53]. In areas with previously undisturbed badger populations, risks were reduced for herds within the proactive zones, but the authors point out that they did not evaluate the effect at the cull edges or within 2 km of the cull. Risk was actually greater in reactive and survey-only areas by 23% and 18%, respectively, indicating that localized reactive culling was associated with a higher risk than not culling, and this was felt at the local farm level. Whether this risk was sustained over time since last cull remains to be reported. Farm and herd size, number of land parcels and contiguous neighbours were the most consistent risk factors, and no consistent risk due to badger- or habitat-related variables was identified at the farm level.

## 7. Summary

In summary, the risk factors that have been most consistently identified in relation to bovine TB, particularly in recent UK and Ireland studies include historic incidence, farm area, cattle movement, occurrence of TB on contiguous premises and/or the level of bovine TB in surrounding areas (infection pressure or force of infection), and herd size (Table 1) [54]. Other factors identified in some studies include indicators of badger density/activity, use of multiple premises, housing type, herd type, farmland habitat, fertiliser usage, mineral deficiencies, and use of silage clamps (Table 2). Herd-keeper behaviour is also likely to change during an outbreak due to increased risk perception, leading to improved biosecurity measures and risk aversion [55].

In general, the most consistently identified risk factors are biologically plausible and consistent with known transmission routes involving cattle-cattle and badger-cattle spread. It is important to note that epidemiological studies differ in the variables analysed, the exact measures used (e.g., in relation to association with badgers), and study size and power. Not all risk factors would be expected to be identified equally across different studies. Risk factors will vary across regions due to factors such as differing farm structures, farm management practices, local TB control, and the relative importance of specific risk factors within individual areas. After extensive and iterative risk factor studies on RBCT data the Independent Scientific Group (ISG) concluded that important risk factors differed between regions and hence other case-control studies of bovine TB in cattle had yielded widely differing recommendations [1].

Taken together, these studies illustrate the complexity of the host/pathogen/environment interactions or “episystem” in bovine TB [56, 57] and the variation in study design and outcome. It may not be possible to reliably identify particular risk factors which could be widely adopted and predicted to lead to reduced transmission of disease to and from cattle. More insight may be achieved when risk factors are classified locally into management, wildlife, and environment factors, [1] and it should be appreciated that environmental features are rarely controllable by the herd keeper. One primary risk factor is cattle density, which increases the probability of transmission *via* aerosol between infectious and susceptible animals [35]. Regarding management factors, results suggest that cattle movements, herd contacts, use of fertilizer, housing, and feeding practices may impact on risk, although study findings identify association and not necessarily causation.

Nevertheless there is sufficient evidence that by applying the broad principles of biosecurity it should be possible to reduce the risk of cattle becoming infected by other animals, including wildlife. Account should be taken of cattle movement on and off the premises, minimising contact with other cattle and between cattle and wildlife and taking greater care with animal housing and feeding practices. In particular, studies, such as, the TB99 and CCS2005 analyses of the GB RBCT data indicate that there is no universal solution for farm management to reduce the risk of a herd breakdown. Occasionally a clear cause and effect relationship can be demonstrated by epidemiological studies, but in most cases the situation is more complex and the research tells us what factors are important concerning a specific question or a theoretical level of risk associated with a particular event, behavior, or contact. While many of the general risk factors for the introduction and spread of bovine TB have been identified, less is known about the practical measures that farmers can take to minimize their risk and the possible impact of common biosecurity practices.

## 8. Methodology

We searched systematically on-line resources (PubMed, Web of Science) to find appropriate peer-reviewed literature and relied on previously identified key publications. Literature was accessed until October 2011, inclusive. We purposefully selected publications that were judged most relevant for the review, with a preference for high-quality systematic reviews. Whilst publications in the last 10 years were favoured we did not exclude highly regarded older publications.

Searches were conducted using combinations of the following key words: “bovine”, “tuberculosis”, and “risk”. In addition, open-access DEFRA R&D project web-pages were searched.

DEFRA web-pages on bovine TB, Phillips and others [58], and the Final Report of the Independent Scientific Group [1], “*Bovine TB: the Scientific Evidence*” were referenced throughout.

## Figures and Tables

**Table 1:** The most consistently identified herd-level risk factors for bovine TB.

Herd-level risk factors most consistently identified^∗^
Cattle movement (estimated to contribute <20% in some GB and
Irish studies)
Occurrence of TB on contiguous premises and/or level of TB in
surrounding areas (infection pressure)
Herd size

^∗^It is important to note that epidemiological studies may differ in the variables examined, the exact measures used (in relation to the association with badgers etc.), and the study size and power. Therefore, not all risk factors would be expected to be identified equally across all studies.

**Table 2:** Other herd-level risks identified in some studies.

Other herd-level risk factors identified in some studies^∗^
Contact with contiguous cattle
Indicators of badger density/activity
Sourcing cattle from herds with TB history
Providing cattle feed inside housing
Use of multiple premises
Housing type
Herd type
Farmland habitat
Fertiliser usage
Mineral deficiencies (selenium)
Use of silage clamps
Rotational grazing

^∗^It is important to note that epidemiological studies may differ in the variables examined, the exact measures used (in relation to the association with badgers etc.), and the study size and power. Therefore, not all risk factors would be expected to be identified equally across all studies.
